# Psychosocial Stresses and Concerns of People Living in Tehran: A Survey on 6000 Adult Participants 

**Published:** 2018-04

**Authors:** Ahmad Ali Noorbala, Hassan Rafiey, Fardin Alipour, Amir Moghanibashi-Mansourieh

**Affiliations:** 1 Psychosomatic Medicine Research Center, Imam Khomeini Hospital, Tehran University of Medical Sciences, Tehran, Iran.; 2 Social Welfare Management Research Center, University of Social Welfare and Rehabilitation Sciences, Tehran, Iran.; 3 Social Welfare Management Research Center, Department of Social Work, University of Social Welfare and Rehabilitation Sciences, Tehran, Iran.; 4 Department of Social Work, University of Social Welfare and Rehabilitation Sciences, Tehran, Iran.

**Keywords:** *Psychosocial Stress*, *People*, *Stressor*, *Tehran*

## Abstract

**Objective:** Nowadays stress and tensions are among the most important factors affecting health. Identifying the stressors and their determinants provides substantial information for understanding the health of the community.

**Method** **:** This descriptive cross-sectional study was conducted on citizens over 18 years who were living in all 22 districts of Tehran in 2017. The participants were selected using multistage cluster sampling method. The research tool was a checklist that evaluated various factors. Different statistical tests, such as descriptive tests and logistic regression, were used for data analysis.

**Results:** Of the participants, 82.7% experienced at least 1severe stress during the past year. In the last year, 45.6% of the participants had at least 1severe economic stress, 32.3% had at least 1severe family-related stress, 28.8% had at least 1severe health-related stress, and 25.7% experienced at least 1severe future-related stress. The most common psychosocial stressors experienced in the last year were concerns about personal/family future (53.7%), concerns about the financial and economic future (47.1%), and the high cost of living (41.7%). However, the most severe stresses were due to the participants’ concerns about family health (14.4%), personal/family futures (13.2%), and financial and economic future (12.7%). Furthermore, health status, subjective socio-economic status, and age were the most important predictors of severe stress experiences.

**Conclusion: **It is necessary to take actions to reduce the prevalence of common severe stresses. In addition, psychologists, psychiatrists, counselors, and social workers need to provide stress management interventions carefully to their patients.

Stress is one of the most common problems in human life that has been under vast investigation in the last century as one of the risk factors to health(1, 2). Hans Selye (1936) was the first to show the importance of stress in mental health and elaborated on the emergence of a "general adaptation syndrome" due to intense psychological pressures(3).

Stress refers to the “physiological or psychological response to internal or external stressor”, which in turn is

 “any internal or external event, force, or condition that requires adjustment or coping strategies on the part of the affected individual.”(4). 

Extensive research has been done on the effects of stress, suggesting that stress is associated with cardiovascular diseases(5, 6), musculoskeletal disorders(7, 8), hypertension(9), and digestive diseases(10).

In addition to the effects of stress on physical health, mental and social health of individuals is also affected by stress(11, 12).

In this regard, different researches demonstrate a significant relationship between stress and suicide(13), burnout(14), post disaster disorders (12, 15), adolescent happiness(16), substance abuse(17), domestic violence(18), and quality of life(19).

In addition to the direct effects of stress on health, reports suggest an extensive financial burden that stress imposes on societies. For instance, the costs associated with stress-related disorders in the United States is estimated to be more than 42 billion USD a year. It is also reported that people who suffer from stress-related disorders are 3 to 5 times more likely to be examined by a doctor than others, and 6 times more likely to be admitted to hospitals(20).

Based on the stimulus-based theory, stress is interpreted as a stimulus, event, or group condition that may create psychological responses and increase individual vulnerability to disease. In this model, the positive and negative events of life are considered as stressful(21). Lazarus has developed theory of cognitive appraisal in defining stress. Based on this theory, stress perception is performed in 2stages of a primary appraisal (assessment or judgment of an event) and a secondary appraisal (assessment of available resources to match that event). He considers stress as the result of an imbalance between the individual's desires and individual’s resources to deal with the event. Lazarus argued that the stress was significantly different between individuals depending on how they interpret an event(22).

 general strain theory, which is among the theories of social deviance, Assumes that in societies with a high level of individual and social stress, where no program is set to prevent and reduce these risk factors, the threshold of individuals’ tolerance is low and this can lead to social deviances, such as violence, addiction, abusive sexual behavior, and crime(11, 23).

Policy- making and planning in mental and social (psychosocial) health require specific attention to psychological and social stress among people. Identification and awareness of the status of psychosocial stressors is the first step in developing a stress management program. In this regard, many studies have been conducted to identify and monitor stressors in different countries. A survey in the United States shows that people are more stressed after the 2016 US election (from 4.8 to 5.1 on a 10-point scale). The findings also suggest that 3factors of economy, terrorism, and mass shoot/gun violence have been the main factors that have increased the level of stress among Americans over the past decade(24).Another study conducted in Thailand in 2010 revealed that women were more stressed than men and that those over the age of 60 had experienced stress more than the younger ones(25).

A further study in Australia in 2014 was conducted among people over the age of 18. The results showed that among the 13 lists of stressful sources, the most common sources of stress are personal financial issues (49%), family issues (45%), and personal health issues 42%), and the least prevalent was issues around personal safety (14%)(26). Findings from a study by Blendon et al. (2014), which was conducted on 2500 people aged 18 and over, showed that around half of the participants (49%) experienced at least a severe stressful event during the previous year. The most common severe stressors were health-related problems (43%), occupational problems (13%), changes in life such as relocation (9%), familial problems such as issues with children (9%), and personal relationships such as divorce (6%)(27).

In Iran, little research has been performed in this area and among the general population. A study in 2008 was conducted on 600 people over the age of 18 in Tabriz. The results suggest child's death, husband's death, and betrayal of the spouse as the most important stressors(28). The results of the study by Malakouti et al. also introduced family members’ health issues, births, unexpected deaths, financial problems, increase of living expenses, unemployment, and family conflicts as the most common stressful events in city of Zahedan in 1998(29). The results of another study in Iran (2011) showed that the 12- month weighted prevalence of any psychiatric disorder was 23.6%, with 26.5% of women and 20.8% of men having one or more psychiatric disorder(30).

By reviewing studies on stress, it seems that despite the extensive costs involved in coping with stress-related diseases, the diverse sources of psychosocial stress have not yet been completely recognized and there is a need to conduct a holistic research to identify psychosocial stressors in the general population. In addition, the identification and observation of stressors annually can provide a better understanding of the state of stress in the community and provide the basis for policy-making and scientific evidence-based planning in this area. Therefore, considering the importance of this issue, the present study tried to study psychosocial stressors in the city of Tehran for the first time in terms of prevalence and severity among the Tehran citizens in 2017.

## Materials and Methods

*Participants and Study Design*

This was a descriptive, cross-sectional study among citizens over the age of 18 living in all 22 districts of Tehran in 2017.For maximum variance ${(\sigma }^{2}=0.25)$ ${(\sigma }^{2}=0.25)$, 95% confidence interval $\left(z_{1-\frac{\alpha }{2}}=1.96\right)$, and 0.05 absolute error, a sample size equal to 385 is needed for any level of analysis. This size was increased by 15% (i.e., 443) to compensate for the probable attrition and by another 50% (i.e., 665) toassume1.5 design effect because of our clustered sampling. Hence, 6000 people were included in the study based on3levels of socioeconomic status (high, middle, and low and 3 age groups (young: 18-39 years old, middle-aged 40 to 59 years, and elderly 60 years and older). Both sexes (men and women) were included in the study.

The method of sampling was multistage cluster sampling. First, the size of the sample was determined proportional to population size (PPS) from each of 22 districts in Tehran. In the next stage, noting that every district of Tehran has social and economic diversity, each district was divided into 3categories of high, medium, and low, based on the housing price and qualitative interview with local trustees. Then, in each district of Tehran, 3neighborhoodswithdifferent economic and social level were randomly selected. In other words, sampling was done in 22 districts and 66 neighborhoods of Tehran. In each neighborhood, among the residents of that neighborhood, the sample size was selected proportional to the sex and age composition of the residents, and the research checklist was completed.

*Measures*

The research instrument was a checklist for gathering information about manifest events in the last 12 months of the participants’ lives, and not a “questionnaire” for a “latent trait” requiring validation studies. The checklist consists of 7 sections: individual events, family issues (for married people), financial and economic events, occupational and working events (especially for the employed), educational events (student-specific events), community events, and future related events. In total, this checklist questioned 159 events that caused stress. Respondents first marked the most common events that have occurred to them in the last year which caused stress, and in the next step, they marked the most severe cases among the items marked in the first section.

This checklist was prepared in several stages: first, to provide a list of stressors, a comprehensive overview of existing scientific resources was performed, which included various tools, such as Holms and Rahe's stress scale questionnaire(31), the burden of stress study in US(27), and the list of psychosocial situations among clients of counseling centers of the state welfare organization of Iran. Subsequently, with 4focus group discussions in 4districts of Tehran, the stressful experiences of citizens were added to the checklist. Next, a list of psychosocial stressors that was extracted from 2previousstageswas provided to 27 mental and social health professionals (psychiatrists, psychologists, social workers, and sociologists), and their views were also applied. In the final stage, to determine the feasibility of the implementation of the checklist and its understandability, a pilot study was performed on 50 citizens. In this phase, it was evident that participants could response easily to the checklist in about 20 minutes, and wordings of the items that were ambiguous for at least to 1 respondent were corrected. The final checklist was prepared for measuring psychosocial stresses and concerns in Tehran.

In qualitative part of the study, the frequent contents that emerged from focus group discussions were “concerns”, which were not predicted based on the definitions of stress and previous works reviewed. To cover this emergent concept in our study, we added these concerns to our checklist. Although it is quite obvious that concerns are about the future, while stress is a response to the events that have occurred before. In this regard, the checklist, and therefore the study, could be considered a checklist and study on stresses and concerns of people.

*Data Collection*

To collect data, a total of 15 interviewers, all of whom had related educations (psychology, social work, and social welfare management) at the graduate and postgraduate level, received a one-day training on the purpose of the study, the inclusion and exclusion criteria, ethical considerations, and how to complete the checklist. With regards to the selected neighborhoods, each of the trained interviewers completed the checklist to assess stressful social and psychological events for the selected samples after obtaining informed consent from the participants. A team of Ph.D. students (social work and health and social welfare) assisted in monitoring the administration of the checklist. In this study, the main inclusion criteria were as follow: (1) age ≥18and (2) informed consent to participate in study.

*Statistical Analysis*

Data were analyzed by SPSS using appropriate descriptive statistics and logistic regression tests.

## Results

Of the participants in the study, 51.6% were male, 48.9% were married, 29.7% had a bachelor's degrees, more than half (58.2%) were employed, and 16.5% were unemployed (Table 1). Also, more than two thirds of the participants in this research (71%) rated their household expenses equal to household income.

To determine the most common psychosocial stressor events, the frequency of experiencing these events was calculated in the last year. Table 2 demonstrates the prevalence of the most common psychosocial stressful events experienced in the past year. As shown in the table, the most prevalent ranks were concerns for the personal/family future (53.7%), concerns of the financial and economic future (47.1%), and worrying about the high cost of living (41.7%), respectively. Corruption was ranked eighth among the psychological stresses(32.3%)and internet related problems was ranked 10^th^(29.4%).At the end of this list were illnesses, injuries, and disabilities or problems in the physical or mental health of the family members (24.6%), concerns about the possibility of accidents and man-made disasters (24.8%), and poor socioeconomic status of the neighborhood (25.1%).Subsequently, participants were asked to choose the most common mental pressures experienced in the last year that caused severe stress. Findings from this section showed that 82.7% of the participants in the study experienced at least 1 severe stress during the past year.

Table 3 shows the findings of the psychosocial stressful events that led to intense stress among participants in this study. The results indicated that the highest level of stresses were associated with concern about the future. Respondents reported that the most important events that caused severe stress were concerns about health of family members (14.4%), uncertainty about personal/family future (13.2%), and concerns about the financial and economic situation in future (12.7%) (Table 3). The results also revealed that 45.6% of the participants had experienced at least 1severe economic stress, 32.3% had at least 1severe family-related stress, 28.8% experienced at least 1severe health related stress, and 25.7% had at least 1severe stress related to the future.

Logistic regression test was used to determine the underlying factors for the variable experience of at least 1 severe stressful event in the last year (Table 4). The results are presented in Table 4, which show that the current health status, subjective socioeconomic status, and age were significant predictors for those with at least 1severe stressful event in the last year. In other words, people with long-term illnesses had experienced higher levels of stress compared to the healthy individuals (p <0.001, OR = 1.55). Furthermore, those with a higher subjective socioeconomic status had lower levels of stress compared to other groups (p <0.001, OR<1). In addition, lower levels of stress were observed in higher ages (p<0.001, OR<1). Overall, the variables entered in the model explained about 9.7% to 17% of the variation of the criterion.

**Table 1:** The Demographic Variables of the Participants

**Demographic Variable**	**Category**	**Frequency**	**Percent**
Gender	Male	3097	51.6
	Female	2903	48.4
Marital status	Single	2684	45.5
	Married	2882	48.9
	Divorced	203	3.4
	Widowed	125	2.1
Education level	Illiterate	78	1.3
	Elementary school	182	3.1
	Middle & high school	528	9.1
	Diploma	1581	27.1
	Associate's degree	796	13.7
	Bachelor	1729	29.7
	Master	820	14.1
	PhD	117	2
Employment	Employed	3145	58.2
	Unemployed	889	16.5
	Housewife	627	11.6
	Retired	268	5
	Others	474	8.8

**Table 2:** The Most Common Events that Caused Psychosocial Stress in the Last Year

**The Most Common Events Causing Psychosocial Stress**	**Prevalence**
	**Percentage**
1. Concerns about individual and family future (e.g., fear of sickness, death, addiction, unemployment and other social issues for children or siblings, failure to do personal affairs in the future)	53.7
2. Concerns about financial and economic future (e.g., fear of bankruptcy or future poverty)	47.1
3. Expensive daily necessities	41.7
4. Concerns about the future of employment (e.g., fear of joblessness, being fired, failure to do business responsibilities)	41.3
5. Concerns about health of a family member (father, mother, sister, brother, spouse, child)	39.8
6. Traffic problems	38
7. Market instability	35.7
8. Corruption of police, court, governmental organizations and bodies, municipality and so on (e.g. bribery, embezzlement, nepotism, and injustice)	32.3
9. Expensive housing	29.7
10. Internet-related issues (including filtering, slowness, disconnection, lack of sufficient coverage)	29.4
11. Living in a neighborhood or community where weather, water, and food is unhealthy or it is really crowded	27.2
12. Salary and wage problems	27
13. Watching, reading, or listening to bad news	27
14. Unemployment	26.4
15. Dissatisfaction with the quality of housing	26.4
16. Concerns about the future of neighborhood and community situation (including fear of war, earthquake, famine and so on in future)	26.3
17. Concerns about life after death (resurrection, hell etc.)	25.9
18. Low socioeconomic and cultural level of the neighborhood	25.1
19. Concerns about the occurrence of human-made disasters and incidents	24.8
20. Disease, injury, disability, or any other problems in physical or mental health of the main family members	24.6

**Table 3:** Events Producing Severe Psychosocial Stress in the Last Year

**Events Causing Psychosocial Stress**	**Severe Stress**
	**Percentage**
1. Concerns about health of a family member (father, mother, siblings, spouse, and child)	14.4
2. Concerns about individual and family future (eg, fear of sickness, death, addiction, unemployment, and other social issues for children or siblings, failure to do personal affairs in the future)	13.2
3. Concerns about financial and economic future (eg, fear of bankruptcy or future poverty)	12.7
4. Disease, injury, disability, or any other problems in physical or mental health of the main family members	11.2
5. Concerns about future of employment (eg fear of joblessness, being fired, failure to do business responsibilities)	11.2
6. Unemployment	11
7. Expensive daily necessities	10.3
8. Death of friends and relatives	9.5
9. Expensive housing	8.8
10. Death of main family members	8.7
11. Market instability	8.7
12. Being far from family members	8.3
13. Salary and wage-related problems	7.7
14. Dissatisfaction with face, height, weight and other appearance dimensions or concerns about their inappropriate changes	7.6
15. Tenancy and problems such as rent pressure, landlord troubles, and repeated movements	7.3
16. Dissatisfaction with quality of housing	7.2
17. Having a major debt	6.9
18. Disease, injury, disability or problem in your physical or mental health	6.8
19. Reaching age of marriage (eg, waiting for a good suitor or family pressure for marriage)	6.6
20. Traffic problems	6

**Table 4:** Predictors of the Experience of at least One Severe Stressful Event in the Last Year

**Predictor Variables**	**B**	**S.E.**	**Wald**	**Df**	**Sig.**	**Exp(B)**	**95% C.I.for EXP(B)**	
							**Lower**	**Lower**
Current health condition (reference group: healthy)			8.546	3	0.036			
Acute disease	0.305	0.160	3.628	1	0.057	1.357	0.991	1.857
Chronic disease	0.443	0.174	6.462	1	0.011	1.558	1.107	2.193
Disability	0.197	0.403	0.238	1	0.626	1.218	0.552	2.684
Perceived socioeconomic status(reference group: Level 10)			19.190	9	0.024			
lower level (1)	0.665	0.368	3.270	1	0.071	1.944	0.946	3.996
Level 2	0.849	0.363	5.456	1	0.019	2.337	1.146	4.766
Level 3	0.994	0.352	7.979	1	0.005	2.703	1.356	5.390
Level 4	1.118	0.349	10.238	1	0.001	3.057	1.542	6.062
Level 5	0.890	0.344	6.716	1	0.010	2.436	1.242	4.775
Level 6	0.958	0.351	7.464	1	0.006	2.606	1.311	5.182
Level 7	1.092	0.364	8.984	1	0.003	2.981	1.459	6.088
Level 8	1.275	0.397	10.312	1	0.001	3.580	1.644	7.797
Level 9	1.219	0.504	5.861	1	0.015	3.385	1.261	9.085
Age	-0.010	0.004	8.775	1	0.003	0.990	0.983	0.996
Constant	2.221	0.445	24.878	1	0.000	9.213		

## Discussion

The most common psychosocial stressors and concerns for Tehran's adult citizens in the year 2017 were concerns about the personal/family future, concerns about the financial and economic future, and worrying about the high cost of living. Furthermore, the most severe stresses and concerns were in the four domains of health, family, economics, and future. In addition, significant predictors of severe stress were the current state of health, subjective socioeconomic status, and age.

Although our findings showed that future health concerns were the most common psychosocial stressful events, the study by Malakouti et al. (1998) in Zahedan explained that hospitalization of the family members, birth, sudden unexpected deaths, financial problems, rising living costs, unemployment, and family disputes were the most common stressful events in life of adults in Zahedan (29).

A study by Blendon et al. (2014) in the US showed that the most stressful factors were health problems, occupational problems, changes in life such as relocation, family problems such as problems with children, and personal relationships such as divorce(27). Moreover, findings of another study in the US revealed that the economy, terrorism, and mass shoot / gun violence were issues that have raised the level of stress in the United States over the past decade(24). A research in Australia (2014) has reported that the most common stressors were financial issues, family issues, and personal health issues(26).Cultural and social differences can be assumed as a strong cause for differences in common stressors in different societies. Despite these differences, it seems that concerns about economic and health issues is one of the most common stressful events in different societies. Based on Lazarus theory, the imbalance between the expectations and the resources of a person to deal with the event causes stress. On this basis, the economic instability of societies and the inability of the individuals to adapt to it can be the reasons for the economic stressors. The theory of stimuli can also be used in explaining health-related stress. In fact, the disease is a stimulus that causes stress, irrespective of cultural issues(21).

The present study showed that concerns about the health of the family members, anxiety about the future of personal/family life, and concerns about the financial and economic future have caused the most severe stresses in participants. Along with these findings, a study in the US reported that health-related issues are at the top of the list of severe stressful events(27). In this research, concern about personal/family future is ranked second in severely stressful events. In another study by Malek et al. (2008) child death was described as the most severe stress factor(28).

In addition to cultural and social differences, it is important to note that in the study by Malek et al. (2008), research questions have been asked as hypothetical pictures of stressful events, and participants have been asked to describe their perception of the stress caused by this. However, in the current study, the participants were asked about objective and actual stressful events.

Findings from our study shows that 82.7% of the research participants experienced at least 1severe stress during the past year, while according to a similar study in the US, 49% of the general population experienced severe stress during the past year(27), indicating a comparatively high level of psychosocial stress among the citizens of Tehran. Previous studies indicated that in societies with a high level of individual and social stress, where there is no program to prevent and reduce these risk factors, the threshold of tolerance is low, and this can lead to social deviations (social harms), such as violence, addiction, abnormal sexual behaviors, and crime(11, 23).

Numerous studies have shown that SES is an important predictor of a wide range of health and disease outcomes. Consistent with the results of the study by Blendon et al. (2014), we found that people who had a higher level of SES, experienced lower levels of stress.

Stresses due to face dissipation, height, weight, and other apparent dimensions in the list of extreme stressful events can indicate the growth of individuality and self-interest, which is the consequence of modernization era. Studies have shown that higher dissatisfaction with an individual's image of his/her body causes higher risks to one’s mental health(32, 33).

The initiative of this study was focused on future stressful events, which also showed to be a major stressor for common and severe stresses. In previous questionnaires and studies, only stressful events have often been asked, while the findings from our focus group discussion emphasized the need to add “concerns” to the list of stressors. Although only 6 items about concerns were listed at the bottom of the checklist, results showed that these items were the main issues in prevalence and severity.

Therefore, it is important to address this issue in future studies and analyses. In addition, it seems that the welfare and social security system in Iran should be further strengthened and expanded to make individuals able to overcome such concerns.

In this study, we adapted the concepts and components of the Holmes and Rahe Stress Scale and tried to resolve its weaknesses. One of the known weaknesses for the Holmes and Rahe Scale and many other stress measurement scales is that the hypothetical event list is assessed.

In these methods, the individual is asked to prioritize the hypothetical events that may be stressful to him/her, while in this study, the stress checklist was based on the actual experiences of the individuals in the last year. Therefore, the results of this study provide a more objective judgment on the state of stressful events.

In addition, most of the scales merely examine the evaluation of stressful events in the past and present. However, in this study, the stressful events of the future are also examined. As previously mentioned, this was the section added to the checklist based on focused group discussions with citizens. In addition, one of the strengths of this study, compared to other similar national and international studies, is the large sample size that contributes to the generalizability of the results of the research. Given that the study was conducted on individuals over the age of 18, the findings did not address the stressors of lower age groups. Hence, it can be of advantage to investigate the stressors in the groups under 18 years. In addition, as a comprehensive checklist of stressful events was provided in this study, a study with a similar checklist at the national level can be performed at different time intervals so that the changes in these factors can be examined further.

Regarding the prevalence and severity of stress in Iran, it is necessary to study the factors related to the experience of stressful events, such as underlying factors and stresses, in detail.

## Limitation

The study has several limitations. First, the lack of prior research studies on the topic, second, the study was limited to Tehran. Therefore, the study findings may not be generalizable to other parts of Iran. However, the present research was among first studies that investigated the prevalence of stressful events in Iran. Future studies should consider the prevalence of stressful events in other cities of Iran. 

## Conclusion

Our study results revealed that more than 80% of Tehran citizens experienced at least 1severe stressful event in the last year, and most of these events are in 4areas of health, family, economics, and concern for the future. Considering the destructive effects of stress on quality of life, physical health, and psychosocial health and its financial burdens, Iran’s psychosocial health system should constantly monitor the stressors, identify intervention centers, adopt selective, indicated, and universal prevention programs at individual, family, neighborhood, and community level.
